# Leveraging Swipe Gesture Interactions From Mobile Games as Indicators of Anxiety and Depression: Exploratory Study

**DOI:** 10.2196/70577

**Published:** 2025-06-26

**Authors:** Vibhav Chitale, Julie D Henry, Ben Matthews, Vanessa Cobham, Nilufar Baghaei

**Affiliations:** 1 School of Electrical Engineering and Computer Science University of Queensland Brisbane Australia; 2 School of Psychology University of Queensland Brisbane Australia

**Keywords:** mobile games, swipe gesture, anxiety, depression, mental health, game data, screening

## Abstract

**Background:**

Anxiety and depression are serious mental health conditions affecting millions of people worldwide; however, they are often underdiagnosed due to limited health care resources. Mobile games, with their widespread popularity and availability, offer a unique opportunity to use user-game interaction data for mental health screening.

**Objective:**

This study aimed to explore whether swipe gesture interactions from mobile games can serve as indicators of anxiety and depression symptoms.

**Methods:**

A total of 82 participants played 3 casual mobile games (puzzle, infinite runner, and object slicing games) for 15 minutes each and completed validated measures of anxiety (Generalized Anxiety Disorder-7; GAD-7) and depression (Patient Health Questionnaire-8; PHQ-8). Data were logged for each swipe event, and metrics were computed using statistical measures, yielding roughly 150 metrics per game. Spearman rank correlations were calculated between each metric and GAD-7 and PHQ-8 scores.

**Results:**

Multiple swipe gesture metrics showed significant associations with both anxiety and depression scores. For the puzzle game, mean swipe speed correlated with PHQ-8 (ρ=−0.405; *P*<.001) and GAD-7 (ρ=−0.400; *P*<.001) scores. For the infinite runner game, mean variance in swipe end pressure showed moderate to strong negative correlation with PHQ-8 (ρ=−0.405; *P*<.001) and GAD-7 (ρ=−0.309; *P*=.007) scores. In the object slicing game, minimum swipe start position along the y-axis correlated positively with PHQ-8 (ρ=0.368; *P*<.001) and GAD-7 (ρ=0.370; *P*<.001) scores.

**Conclusions:**

The findings from this exploratory study provide preliminary evidence supporting the feasibility of using swipe gesture interactions in mobile games as novel, engaging, and nonintrusive indicators of anxiety and depression.

## Introduction

### Background

Could a simple swipe interaction during mobile gameplay reveal hidden insights into our mental well-being? In this digital age, mobile phones have become ubiquitous and have engaged billions of people worldwide in mobile gaming activities. These activities generate vast amounts of data on user-game interactions that may offer insights into our underlying mental health. If these data can be harnessed purposefully, mobile games could serve as a widely accessible tool for screening symptoms of mental health disorders such as anxiety and depression. Symptoms of anxiety and depression are a global concern, affecting millions of individuals worldwide [1,2]. However, these conditions often go undiagnosed or untreated due to factors that include resource shortages, lack of trained professionals, societal stigma, and restricted access [3-6]. In response to these challenges, emerging research has begun to increasingly pivot toward leveraging digital platforms such as mobile devices for screening the symptoms of anxiety and depression.

Previous research has examined the relationship between symptoms of mental health disorders and mobile device interactions. Particularly, data derived from smartphone use patterns including touch behavior, keyboard dynamics, and app use can be associated with symptoms of mental health disorders [7-11]. Early studies reported promising associations between typing speed, accuracy, and mood disturbances [12-16]. However, recent literature involving larger sample sizes has yielded mixed results [17]; for example, Ning et al [18] found that keyboard dynamics coupled with accelerometer data from smartphone typing interactions correlated primarily with cognitive performance rather than mood directly. Similarly, Braund et al [17] reported that adolescents with higher anxiety or depression symptoms tended to type slightly faster on smartphones, although this showed weak correlations in a large-scale study. However, Knol et al [19] demonstrated substantial relationships between typing patterns and affective symptoms (eg, anhedonia) in a clinical sample experiencing suicidal ideation. These mixed outcomes highlight the necessity of exploring additional, potentially complementary digital biomarkers.

Particularly, swipe gesture interactions during mobile gameplay present a promising alternative. Mobile games are highly engaging; fun to play; and, by their design, inherently test a variety of cognitive and psychomotor processes such as reaction times or reflexes, sustained attention and focus, hand motor coordination, and quick decision-making through precise touch gesture interactions, all of which can be influenced by anxiety and depression [20]. Given that impairments in these cognitive and psychomotor processes are often associated with symptoms of anxiety and depression [21,22], it seems reasonable to hypothesize that they can be captured through mobile game interactions. However, research on gesture patterns from mobile games and their relationship with symptoms of anxiety and depression remains largely unexplored [23], leaving several important questions unanswered. For instance, which category of mobile games can be effectively used or what game mechanics should be included to elicit game interactions pertinent to symptoms of anxiety and depression? Which gesture or swipe pattern metrics should be tracked? What is the minimum duration of data collection required? These unanswered questions regarding crucial design decisions highlight the need for an exploratory study to establish the foundation for this emerging topic and were the primary motivation behind this work.

To address these questions, we conducted an exploratory study with 82 participants using 3 casual mobile games (CMGs)—a puzzle game, an infinite runner game, and an object slicing game. These games were selected for their simplicity, while also being distinct enough in their design and swipe interactivity to allow for the examination of a range of cognitive and behavioral responses. This simplicity ensures that the games are accessible to a broad audience while potentially minimizing the effect of confounds such as previous gaming experience or gaming skill. As there is no consensus regarding specific swipe or gesture metrics, we adopted a broad approach by collecting a wide range of swipe gesture attributes including swipe speed, swipe positions, pressure applied during the swipe, and more.

The primary aim of this exploratory study was to determine whether subtle swipe gesture interactions during mobile gameplay can serve as behavioral biomarkers of anxiety and depression symptoms. Specifically, we examined correlations between standardized mental health questionnaires such as Generalized Anxiety Disorder-7 (GAD-7) and Patient Health Questionnaire-8 (PHQ-8) and a broad range of swipe metrics. Our findings revealed significant associations between several swipe data metrics and symptoms of anxiety and depression. Key metrics such as swipe speed, swipe pressure variability, and swipe starting positions were significantly correlated with both anxiety and depression scores across the 3 games. These results suggest that subtle patterns in swipe gesture interactions during mobile gameplay can possibly provide meaningful insights into an individual’s mental health. The findings from this study establish a foundational groundwork that speaks to the potential of mobile games as a novel, scalable, accessible, and highly engaging tool to support and enhance traditional methods of screening anxiety and depression.

### Related Works

This research topic is still an emerging field, with fewer than 5% of the studies that have looked at gaming technologies as potential tools for mental health focused on their value in detecting symptoms of anxiety and depression [23,24]. Digital technologies have emerged as promising tools for mental health screening over the past decade [25]. These methods seek to exploit the potential of automatically generated and aggregated data from smartphones, wearables, and other sensory devices to measure digital behavioral markers of mental health conditions [26-30]. Despite these advances in leveraging general digital and smartphone behaviors as digital biomarkers or interventions for mental health, the potential of mobile games remains largely unexplored. Given that this research field is still in its infancy, we first present an overview of the broader literature on mobile games and mobile touch interactions in relation to symptoms of mental health disorders.

Due to the huge popularity of mobile games along with their effects on cognitive functioning and well-being, research on their potential to track symptoms of mental health disorders has attracted growing interest. For example, researchers used a mobile game called Sea Hero Quest [31] to track and assess symptoms of dementia by collecting spatial navigation data from over 4.3 million players. This project remains one of the largest studies on spatial navigation deficits and provided compelling evidence that proved that this cognitive ability begins to decline from the age of 19 years with significant differences between men and women.

Building on the premise of mobile gameplay, Anzulewicz et al [13] demonstrated the potential of using 2 tablet games to detect motor abnormalities in children with autism spectrum disorder (ASD). They analyzed gesture patterns using machine learning and were able to differentiate between children with ASD and typically developing children with 93% accuracy. Motor patterns such as movement kinematics and gesture force were found to be core features of ASD that could be identified using mobile gameplay. The principles behind this study of analyzing gesture patterns can thus be extended in the context of anxiety and depression where motor and other cognitive impairments are also common. For instance, individuals with anxiety often exhibit psychomotor agitation, while those with depression experience psychomotor retardation.

An interesting study by Dechant et al [32] explored the use of games for assessing social anxiety. The gaming task involved participants moving toward a target location and answering an attention test in the presence of nonplayable characters. The key findings revealed that players with higher social anxiety scores tended to stay further away from nonplayable characters and exhibited larger errors in their estimates of the target location similar to avoidance behaviors shown in the real world. Although this study used video games, these results demonstrated that characteristic behaviors associated with social anxiety could also be manifested and tracked in gaming environments.

Recent research has increasingly recognized the potential of mobile games in identifying cognitive difficulties [33-38]. For instance, Intarasirisawat et al [39] conducted a study to explore if the touch and motion features extracted from popular off-the-shelf mobile games such as Candy Crush, Tetris, and Fruit Ninja can be useful for cognitive assessment. They reported several features that were significantly associated with cognitive scores. For instance, an increase in swipe speed was associated with a decrease in performance on cognitive capacity for visual search, mental search, and response inhibition. Other metrics such as swipe length, maximum score, and accelerometer data were also found to be associated with poorer cognitive test scores.

Similar research by Gao et al [40] explored whether touch interactions such as swipe speed and swipe pressure collected from the mobile game Fruit Ninja could indicate the player’s emotional states. They investigated whether variations in touch dynamics were associated with different emotional states during gameplay. They observed that certain touch features such as the pressure metrics could discriminate frustration from excitement, relaxation, and boredom. For instance, they reported that higher pressure often correlated with frustration, and lower pressure was associated with relaxation. Despite the relatively preliminary nature of this research area, these studies collectively demonstrate the potential application of mobile games as a promising platform to analyze touch gesture patterns that could indicate the presence of symptoms of underlying mental health conditions.

## Methods

We conducted an exploratory study in which participants were asked to complete validated measures of anxiety and depression and play 3 CMGs. We aimed to investigate whether there are significant associations between swipe gesture metrics and symptoms of anxiety and depression.

### Recruitment

A total of 82 participants between the ages of 18 to 40 years were recruited from the general population through social media platforms (eg, Facebook and LinkedIn), email invitations, and flyer distributions in the university. Participants were required to be aged at least 18 years with a basic understanding of the English language to complete the required questionnaires. Participants with severe physical or cognitive impairments that limited their ability to interact and play mobile games were excluded from the study.

### Measures

In this study, we used several standardized measures to assess symptoms of anxiety and depression among the participants.

#### The PHQ-8 and GAD-7 Questionnaires

The GAD-7 [41] and PHQ-8 [42] questionnaire were used to measure symptoms of anxiety and depression, respectively. The GAD-7 is a 7-item self-report questionnaire that evaluates the presence and frequency of general anxiety symptoms experienced over the past 2 weeks on a scale range from 0 (not at all) to 3 (nearly every day). The total scores range from 0 to 21, with higher scores indicating greater severity of anxiety symptoms. The GAD-7 is a widely used and validated self-report measure, with high internal consistency (Cronbach α=0.92) and good test-retest reliability [41]. Similarly, PHQ-8 is an 8-item self-report questionnaire that assesses the frequency of common depression experienced over the past 2 weeks on a scale range from 0 to 3, with higher scores indicating more severe depressive symptoms. PHQ-8 is a variant of the PHQ-9 measure, which excludes the ninth item related to suicidal thoughts, making it more suitable for use among the general population. PHQ-8 is well established as a valid diagnostic tool for assessing depression with excellent internal consistency (Cronbach α=0.89) [41].

In our sample, the mean anxiety score was 9.09 (SD 5.26) while the mean depression score was 8.24 (SD 4.95) as indexed by GAD-7 and PHQ-8 questionnaires, respectively. Table 1 summarizes the clinical severity categories.

**Table 1:** Anxiety and depression severity distributions (N=82).

Severity		Participants, n (%)
**Anxiety distribution (GAD-7^a^)**		
	None	23 (28)
	Mild	23 (28)
	Moderate	20 (24)
	Severe	16 (20)
**Depression distribution (PHQ-8^b^)**		
	None	25 (30)
	Mild	25 (30)
	Moderate	23 (28)
	Severe	9 (11)

^a^GAD-7: Generalized Anxiety Disorder-7.

^b^PHQ-8: Patient Health Questionnaire-8.

#### Demographics and Gaming Behavior Questionnaire

Participants were asked to provide basic demographic information including age, gender, and gaming habits. Gaming habits were quantified using questions about gameplay frequency (daily, regular, or occasional gamer); average time spent playing games in one session (short, moderate, or long sessions); and self-identified gamer identity (nongamer, casual gamer, or gamer). Table 2 provides an overview of the demographic and gaming behavior characteristics of participants.

**Table 2:** Participant demographic and gaming behavior information (N=82).

Category			Participants, n (%)
**Sex**			
	Male	31 (38)	
	Female	51 (62)	
**Age groups (y)**			
	18-20	41 (50)	
	21-30	37 (45)	
	31-40	4 (5)	
**Gamer identity**			
	Nongamer	15 (18)	
	Casual gamer	53 (65)	
	Gamer	14 (17)	
**Gameplay frequency**			
	Daily gamer	23 (28)	
	Regular gamer	35 (43)	
	Occasional gamer	24 (29)	
**Gaming session duration**			
	Short gaming session	27 (33)	
	Moderate gaming session	45 (55)	
	Long gaming session	10 (12)	

### Game Design

#### Overview

Due to the diversity of mobile games in terms of genres and mechanics, it is essential to understand which types of game elements hold the most potential to allow for swipe gesture interactions that could indicate symptoms of anxiety and depression. At the same time, it is crucial that these game elements or mechanics are simple enough to reduce the effects of potential confounds such as previous gaming experience or gaming skill. This would minimize the risk of skewing results due to an individual’s familiarity with gaming rather than due to symptoms of anxiety or depression.

Therefore, for this study, we decided to use 3 CMGs: a puzzle game, an infinite runner game, and an object slicing game. CMGs refer to games that are easy to learn and play with simple mechanics involving short gameplay sessions. Some examples of CMG include popular games such as Candy Crush, Subway Surfers, Fruit Ninja, and Angry Birds. These games also have the advantage of being played by a wide range of demographics, which makes it easier to gather data from diverse populations, including people from different ethnicities, age groups, and genders. In contrast, games such as Space Invaders that rely on multibutton taps and often 2-handed controls cannot provide continuous swipe data. Therefore, highly complex games were avoided to minimize confounds from previous gaming skill and to isolate pure swipe behavior. Furthermore, the positive reception and potential benefits of casual games in mental health contexts, as reported by Pine et al [43], suggest that CMGs could potentially enhance user experience and compliance.

To optimize the benefits of games, it is also critical that the games are well-refined and engaging enough to keep players immersed and motivated throughout the gaming sessions. We used the Unity3D game engine to develop our own versions of the 3 games based on the commercially available off-the-shelf mobile games using the available assets from the Unity3D asset store [44-46]. These games were embedded with our custom data collection pipeline that allowed for tracking several metrics related to the swipe behavior patterns as well as the device motion data recorded from the accelerometer and the gyroscope sensors. All games were play-tested before the actual experiment session to identify and resolve bugs within the gameplay and data collection systems.

#### Puzzle Game

**Figure 1:**
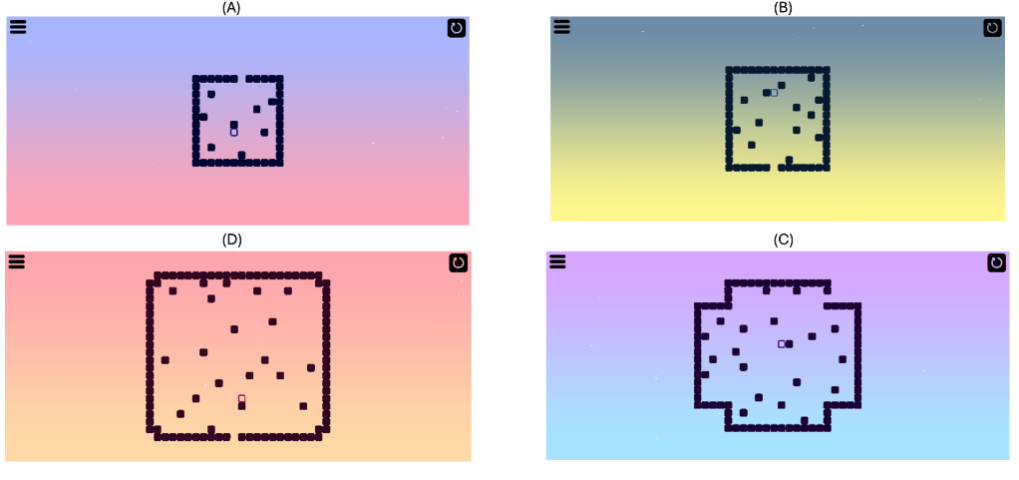
Four screenshots from the puzzle game depicting various 2D levels, with increasing difficulty clockwise from top left. Bottom left shows level 17, the average level reached by participants.

Figure 1 depicts the design of the puzzle game. In this game, the objective is to help the player (transparent square block) escape the maze of square blocks from the designated way out (blank space in the outer blocks). The interactions are extremely simple with only swipes in 4 directions (up, down, left, and right), moving the player in the corresponding swipe direction. The player continuously moves in the given direction until it hits another block or any of the outer blocks. The player’s direction cannot be changed while in motion.

A retry button (top right corner button in all images from Figure 1) was provisioned to reset the level if users were stuck completing a level, which simply put the player back to the original starting position when the current level was loaded. There was no time limit or retry limit for each individual level, except for the total time limit of 15 minutes for the current game session duration. The puzzle game was played in a landscape orientation of the smartphone. We chose a puzzle game genre because it places demands on various cognitive abilities such as visuospatial processing, working memory, and information processing speed [47-49]. Completing levels demands attentional focus and strategic planning and, at the same time, failure to complete could trigger frustration or irritation. We believe that symptoms such as frustration, visuospatial capabilities, ability to maintain focus, or irritability and hesitation could be manifested within such a type of gameplay. The design also incorporates levels with increasing difficulty as the game progresses, as research has shown this type of design keeps the players motivated and engaged with the gameplay for a longer duration [49].

#### Infinite Runner Game

**Figure 2:**
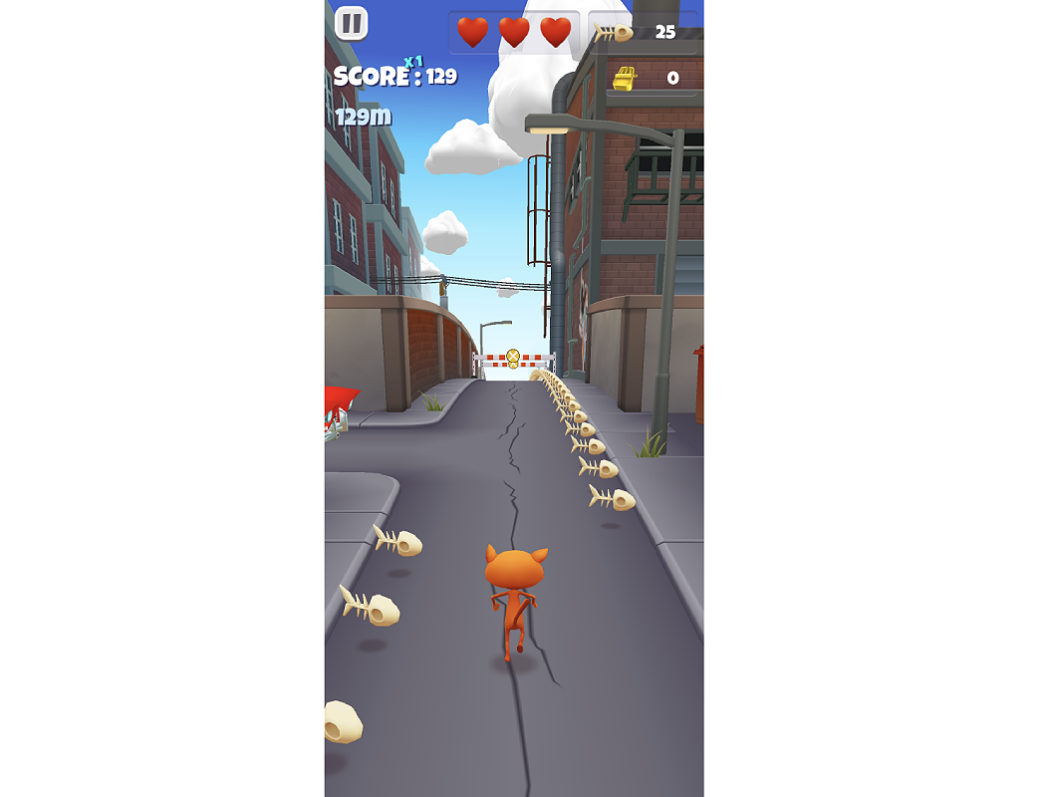
A screenshot of the 3D infinite runner game depicting the player character (orange cat) with collectibles (fishbones) and obstacles. The top right corner displays total items collected and the top left corner displays the total score and distance covered.

Figure 2 shows the design of the infinite runner game. The gameplay is a clone of the popular infinite runner game called Subway Surfers. In this game, the task is to navigate the player character (orange cat) that automatically moves forward through an endless environment filled with obstacles, collectibles, and power-ups. The users control the player character using only swipes in 4 directions that correspond to different player actions. The “up” direction swipe causes the player to jump over obstacles, while the “down” direction swipe slides the player below the incoming obstacles. The left and right swipes move the player in the left or right direction, respectively. The speed of the player moving forward gradually increases as the game progresses, with collectibles and power-ups assisting the player to boost their score and overcome obstacles. The player receives 3 lives (depicted as 3 hearts at the top middle portion of the screen in Figure 2) in each round before eventually reaching the game over status. Every participant is allowed to play as many rounds as they can complete within a total duration of 15 minutes. The infinite runner game was played in a portrait orientation on the smartphone.

This specific design was chosen because the gameplay demands sustained attention, quick reflexes, and precise hand motor coordination to make the appropriate swipes for dodging the incoming obstacles. In addition, players need to successfully obtain collectibles (fishbones), premium valuables (gold bars), and power-ups to boost their score, challenging their working memory capacity by requiring them to keep track of several things at once. Moreover, infinite runner games have been previously shown to be effective in tracking symptoms of mental health disorders [33,50,51].

#### Object Slicing Game

**Figure 3:**
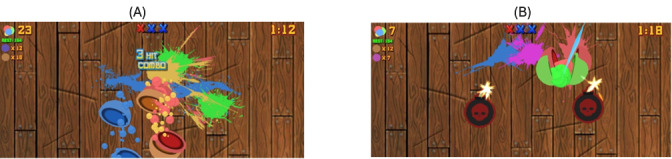
Two screenshots from the object slicing game with the left image depicting slicing of objects while the right image shows 2 bombs that should be avoided. The screenshots also display several user interface elements such as the 3 crosses in the top middle portion of the screen.

Figure 3 shows the design of the object slicing game. The game was developed as a clone of the popular Fruit Ninja game with a few modifications. The game requires the user to swipe the device screen to slice the objects that appear. The goal is to slice as many objects as possible within a time limit of 90 seconds for each round. If the users miss 3 objects (depicted as 3 crosses at the top middle portion of the screen in both images in Figure 3) or if a bomb is sliced (right image in Figure 3), it results in a game over status. Every participant gets to play as many rounds as they can complete within a total duration of 15 minutes. This game was played in a landscape orientation on the smartphone.

This game was chosen as it involves participants to carefully execute swiping gestures to ensure only objects are sliced while avoiding the bombs thrown in the mix, which requires precise gestures and hand-eye-coordinated movements. Unlike the previous two games, this game does not involve swipe gestures in any particular direction but rather requires users to make longer and continuous swipes. These types of interactions have been previously shown to be sensitive to cognitive difficulties and frustration [39,40]. We believe the addition of time pressure and game over threat from missing objects will increase its sensitivity to symptoms related to flight-or-fight responses, frustration, fatigue, and motor abnormalities within the swipe gesture patterns of the participants.

### Ethical Considerations

This study was approved by the University of Queensland Human Research Ethics Committee A (2023/HE000853). Participants were first given a brief introduction to the experiment and asked to provide written informed consent before commencing the study. Adherence to ethical guidelines, participant privacy, safety and data integrity were prioritized at every stage of the study. Participants were reminded that their participation was voluntary, and they were free to withdraw from the study at any time. All collected data were encrypted, anonymized and deidentified before conducting any analyses. Compensation for participation was offered in the form of either course credits or an AUD $10 (US $6.50) gift card voucher.

### Procedure

#### Overview

**Figure 4:**
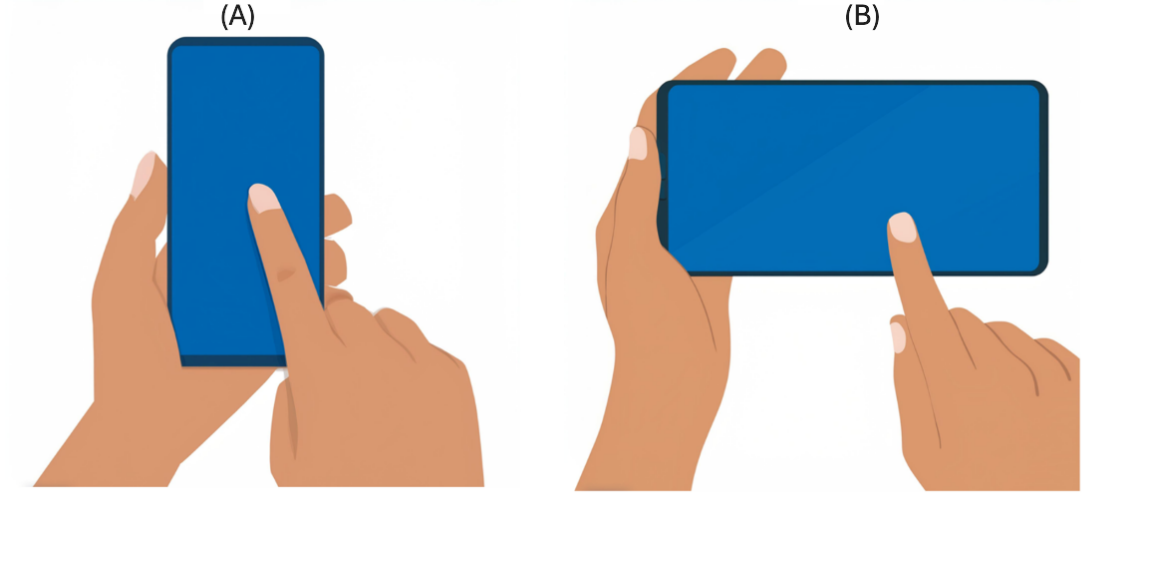
Protocol to hold the phone in one hand and play the game with the other using single finger touch in two different phone orientations.

After providing consent, participants were asked to provide brief demographic information and respond to gaming behavior questions that asked about their gaming habits, such as their gaming frequency and the average time they spent playing games. On completion, participants were provided an Android Google Pixel 6a smartphone (without a screen protector), which was preloaded with the 3 games designed for the study. Participants were given detailed instructions on the protocol that they needed to follow while playing the games. The participants were advised to play the games while seated on a chair, holding the device in one hand while playing the game with the other hand, as illustrated in Figure 4. They were asked not to place the hand or rest the hand on the table during the gameplay. Furthermore, they were instructed to only use a single touch (ie, only 1 finger was allowed to touch the screen at a time) while playing the games. Finally, participants were encouraged to play the games as they normally would while abiding by the protocol.

The rationale for asking participants to strictly follow the protocol was to ensure consistency and reduce the influence of extraneous factors such as variations in hand size and finger length that can influence the collected data and potentially lead to false associations. For instance, differences in the hand and finger measurements can affect how participants interact with the smartphone, thereby influencing swipe metrics such as swipe speed or touch radius. By adhering to a standardized protocol, these variations were controlled, helping to ensure that any individual variance observed in the collected swipe gesture metrics is more likely to reflect differences in underlying psychomotor and cognitive processes rather than anatomical differences. Therefore, this standardized protocol helps minimize potential confounds, allows for replicability, and enhances the validity of the study’s findings.

Thereafter, participants were asked to complete a tutorial session of approximately 10 minutes, in which they played games like the ones used in the study. The purpose of this tutorial session was to allow the participants to become comfortable with the size and shape of the smartphone while familiarizing themselves with the protocol and the gameplay. The tutorial session also aimed to reduce any potential biases related to previous gaming experience or varying skill levels among the participants before the actual data collection. Upon completion of the tutorial session, participants were randomly allotted to 1 of 2 groups. Participants in group 1 played the mobile games first and then completed the anxiety and depression questionnaires, while the participants in group 2 completed the questionnaires first and then played the games. In addition, the order of the games and the questionnaires was randomized within each group. This randomization and counterbalancing were implemented to reduce any potential biases that could arise from the sequence of activities. Participants played each game for 15 minutes, with the entire experimental session lasting around 120 minutes. An instructor was present during the gameplay session to ensure participants adhered to the gameplay protocol and to monitor for any signs of disengagement or noncompliance, but all participants completed the mental health questionnaires privately.

#### Data Collection

The swipe gesture data collection occurred in real time during the gameplay session. Because of limited knowledge with respect to which metrics to track, we used a broad data collection approach, designed to capture a wide range of data metrics in real time. As overcollection of data could result in lags or framerate drops (ie, temporary reductions in the game’s rendering rate causing choppy visuals, game stutter, and input lag) that are known to negatively impact user engagement and experience, we used event-based data collection. Because the main interactions with mobile games were using swipe gestures, we opted to log data entries whenever a swipe event occurred within the game along with a timestamp. This resulted in a large volume of datasets, rich with patterns of swipe gesture behaviors. For example, the entire raw dataset included >60,000 entries only for the puzzle game. Table 3 lists the swipe gesture metrics that were collected during the gameplay. The collected data were stored on the device locally as JSON (JavaScript Object Notation) files before it was transferred to the server. A unique user ID was assigned to each log file as well as the questionnaires to link them. A visual depiction of the swipes from each of the 3 games is provided in Multimedia Appendix 1.

The primary categories of data metrics included swipe behavior metrics (swipe speed, swipe direction, swipe duration, swipe pressure, etc). The swipe positions were stored separately for individual X and Y components, resulting in additional metrics corresponding to the x-axis (swipe_posX) and y-axis (swipe_posY). Due to hardware limitations that currently restrict direct measurement of touch screen pressure, the swipe pressure was quantified using the size of each participants’ fingertip contact area with the screen as a proxy for the pressure. This approach is a common practice in research involving touch-based interactions and has been validated in previous research as a reliable approximation [40,51]. In addition to the swipe gesture metrics, the device sensor metrics were also recorded from the accelerometer and gyroscope sensors, independently of the swipe events.

**Table 3:** Swipe gesture metrics collected from all 3 games.

Metric	Description
swipe_ID	The swipe event identifier
swipe_event_time	Time in seconds from the game launch when this swipe event occurred
swipe_start_time	Time in seconds from the game level when this swipe started
swipe_end_time	Time in seconds from the game level when this swipe ended
swipe_start_pos	The x-axis and y-axis coordinate positions of the screen touch when swipe started
swipe_end_pos	The x-axis and y-axis coordinate positions of the screen touch when swipe ended
swipe_pos	A list of all x-axis and y-axis coordinate positions for the current swipe
swipe_start_press^a^	The pressure applied to the screen when swipe was started
swipe_end_press	The pressure applied to the screen when swipe was ended
swipe_start_press_variance	The variance in the pressure when the swipe was started
swipe_end_press_variance	The variance in the pressure when the swipe was ended
swipe_press	The average pressure applied for the duration of the current swipe
swipe_press_variance	The overall variance in the pressure for the duration of the current swipe
swipe_speed	The speed of the current swipe
swipe_duration	The total time in seconds it took to complete the current swipe
swipe_distance	The total distance of the screen covered during the current swipe
swipe_direction^b^	The direction in which the current swipe was performed
time_between_swipes	The time in seconds that elapsed since the previous swipe

^a^press: touch pressure.

^b^Swipe direction was calculated only for puzzle and infinite runner games.

#### Data Analysis and Preprocessing

Before the analysis, data preprocessing steps were carefully planned and implemented to ensure that the data were clean, of high quality, and suitable for subsequent analyses because of the large amounts of data collected. Performing analyses on poor-quality data can compromise the validity of the results.

##### Data Cleaning

Initially, the data were cleaned by removing unwanted attributes or incorrectly logged entries that were collected. Incorrect swipe entries such as swipe entries logged from the main menu interactions before and after the gameplay were removed. This ensured that only the gameplay interactions were included in the final analysis rather than menu navigation metrics. A visual inspection of the data revealed a few unusual swipe entries such as taps on the screen or false touches at the screen edges, possibly due to unwarranted screen contact with participants’ palms. These entries were removed by establishing a minimum threshold distance to differentiate between intentional swipes and unintended interactions. Following the initial data cleaning, outlier detection methods were used to detect and remove outliers from the dataset. This is an important step in the preprocessing phase as outliers can significantly impact the subsequent analyses. The IQR method was used to detect and remove outliers. The IQR method was chosen due to its robustness to various data types, including null entries and zero values, ensuring that the outliers are correctly identified and resolved.

##### Data Aggregation

Because we opted for event-based data collection, our dataset resembles trace data with each individual entry having no meaningful interpretation. For example, a single entry of the recorded swipe metrics such as the swipe position x and y coordinates provides no information about the underlying psychomotor or cognitive processes. However, aggregated data for each user with appropriately engineered features allow us to draw valuable insights. This is because, by aggregating data with engineered features, we transformed the raw event-based data into more interpretable metrics that could potentially indicate symptoms of anxiety and depression.

Therefore, we first aggregated the data for each user across the entire game session separately for all 3 games. This process included summarizing the raw data into more comprehensive statistical metrics to capture the broader forms of gesture patterns. For each game, we extracted several swipe gesture properties (eg, swipe speed, swipe duration, swipe pressure, and swipe start and end positions on x- and y-axes separately). We then aggregated each metric across the 15-minute session using 8 statistical functions (mean, median, maximum, minimum, SD, range, first, and last) wherever appropriate for each relevant metric. This yielded approximately 150 metrics for each game. Some of these higher-order metrics can be challenging to interpret. For instance, “swipe_press_variance_std” represents the SD of the variance in touch pressure values across all swipes performed during the entire game session. It quantifies the inconsistency in how variable the user’s touch pressure is across swipes. Higher values indicate that some swipes are performed with highly fluctuating pressure while others are more stable, suggesting erratic or dysregulated motor control. To ensure transparency and facilitate understanding, a complete list of all the metrics generated along with their plain language definitions has been provided in Multimedia Appendix 2.

##### Data Analysis

Due to the large number of metrics, it was crucial to account for multicollinearity by identifying pairs of metrics that were highly correlated (*r*≥ 0.90). Features that met this threshold were consequently reduced to a single metric. From each pair or group of collinear features, a single representative feature was retained. The choice of retained features prioritized interpretability and relevance, such as preferring SD for assessing variability, rather than combining features mathematically. For instance, “swipe_distance” was excluded in favor of “swipe_speed,” which incorporates both time and distance components and has greater clinical relevance by more directly reflecting psychomotor functioning. This approach ensured the final set of metrics remained informative while reducing redundancy and preserving computational efficiency.

Spearman rank correlation was used to investigate the relationship between the mobile game swipe metrics and symptoms of anxiety and depression as indexed through the standardized measures. Spearman rank correlation was chosen because it is a nonparametric test that does not make assumptions about normality and linearity in the data. For instance, Spearman rank correlation is robust to nonlinear relationships in a dataset because it focuses on the monotonic relationship between variables, meaning it only looks for a consistent trend of increase or decrease between the variables, regardless of whether that trend follows a straight line (linear relationship) or a curved path (nonlinear relationship). As the primary objective was to identify metrics that show potential associations with anxiety and depression scores to inform future confirmatory analyses, we did not apply a correction for multiple comparisons. Therefore, the reported *P* values are uncorrected, and the results should be interpreted as preliminary.

All analyses were conducted using Python programming language (version 3.10.11) and the relevant statistical packages. The primary packages used included *Pandas* (version 2.2.2), *NumPy* (version 2.0.0), *SciPy* (version 1.14.1), *Pingouin* (version 0.5.4), *Matplotlib* (version 3.9.2), and *Seaborn* (version 0.13.2) for visualization and *iTables* (version 2.1.4) for interactive table views in Visual Studio Code.

Due to device labeling errors, the unique “user_ID” assigned to the last 4 participants could not be matched between gameplay logs and questionnaire responses. The corresponding entries for these users were removed, resulting in data from 78 participants contributing to the final analysis.

## Results

### Overview

Spearman rank correlation coefficients were computed to examine the relationship between mobile game swipe metrics with anxiety and depression scores as measured by GAD-7 and PHQ-8, respectively. The results revealed significant correlations, which are summarized in following sections. Due to the large number of data metrics from all the games, only statistically significant results (*P*<.05) are presented.

### Correlation Analysis

#### Puzzle Game Analysis

As shown in Table 4, several puzzle game swipe metrics were significantly associated with PHQ-8 and GAD-7 scores. The variance in the swipe end pressure showed some of the strongest correlations with the PHQ-8 score, with the mean variance (ρ=−0.425; *P*<.001) and the SD of the variance in swipe end pressure (ρ=−0.403; *P*<.001) both moderately to strongly related to depression. Similarly, the average variance in the overall swipe pressure during the entire session was moderately associated with PHQ-8 scores (ρ=−0.326; *P*=.004). Notably, the average swipe speed was also moderately to strongly correlated with PHQ-8 scores (ρ=−0.405; *P*<.001).

In relation to GAD-7 scores, the SD of the swipe end pressure (ρ=−0.334; *P*=.003) and the mean variance of the swipe end pressure (ρ=−0.317; *P*=.006), both showed moderate negative correlations. Furthermore, the average swipe speed was moderately to largely negatively correlated with GAD-7 scores (ρ=−0.400; *P*<.001). The average swipe duration was also positively correlated with GAD-7 scores, consistent with our finding that slower swipe speeds (meaning longer swipe durations) are associated with high anxiety and depression scores.

**Table 4:** Significant Spearman rank correlations between the puzzle game metrics and Patient Health Questionnaire-8 (PHQ-8) and Generalized Anxiety Disorder-7 (GAD-7) scores.

Metric		Spearman rank correlation (ρ)	*P* value
**PHQ-8**			
	swipe_start_press^a^_std	−0.249	.03
	swipe_end_press_mean	−0.237	.04
	swipe_end_press_std^b^	−0.273	.02
	swipe_end_press_last	−0.244	.04
	swipe_end_press_variance_mean	−0.425	<.001
	swipe_end_press_variance_max	−0.278	.02
	swipe_end_press_variance_std	−0.403	<.001
	swipe_end_press_variance_last	−0.292	.01
	swipe_press_variance_mean	−0.326	.004
	swipe_press_variance_min	−0.293	.01
	swipe_press_variance_last	−0.235	.04
	swipe_duration_max	0.258	.03
	swipe_duration_median	0.239	.04
	swipe_speed_mean	−0.405	<.001
	swipe_speed_min	−0.235	.04
	swipe_speed_max	−0.268	.02
	swipe_speed_std	−0.263	.02
	swipe_speed_last	−0.268	.02
	time_between_swipes_mean	−0.249	.03
	time_between_swipes_min	−0.262	.02
	acl^c^_X_std	−0.332	.03
**GAD-7**			
	swipe_end_posX_std	−0.252	.03
	swipe_end_posY_first	0.241	.04
	swipe_start_press_std	−0.262	.02
	swipe_start_press_variance_std	−0.269	.02
	swipe_end_press_std	−0.334	.003
	swipe_end_press_last	−0.238	.04
	swipe_end_press_variance_mean	−0.317	.006
	swipe_end_press_variance_max	−0.260	.03
	swipe_end_press_variance_std	−0.322	.005
	swipe_press_variance_mean	−0.251	.03
	swipe_press_variance_std	−0.248	.03
	swipe_duration_mean	0.269	.02
	swipe_speed_mean	−0.400	<.001
	swipe_speed_max	−0.234	.04
	swipe_speed_median	−0.383	<.001
	swipe_speed_last	−0.242	.04
	time_between_swipes_min	−0.251	.03
	acl_Y_min	0.243	.04
	acl_Y_max	0.252	.03
	acl_Z_max	−0.247	.04

^a^press: pressure.

^b^std: SD.

^c^acl: accelerometer.

#### Infinite Runner Game Analysis

Table 5 reports significant Spearman rank correlations between the infinite runner game swipe metrics and anxiety and depression scores. Negative correlations emerged between several swipe pressure metrics, with the strongest of these between the average variance in swipe end pressure and PHQ-8 scores (ρ=−0.405; *P*<.001) as well as with GAD-7 scores (ρ=−0.309; *P*=.007). Similar to the puzzle game, the mean swipe speed was significantly negatively associated with both PHQ-8 (ρ=0.331; *P*=.003) and GAD-7 (ρ=−0.272; *P*=.02) scores. Interestingly, the SD of the y-coordinate position when starting the swipes was significantly negatively associated with both PHQ-8 (ρ=−0.244; *P*=.03) and GAD-7 (ρ=−0.271; *P*=.02) scores. In addition, the total number of swipes was negatively associated with anxiety (ρ=−0.266; *P*=.02).

Notably, for the infinite runner game, substantial associations were evident between several accelerometer metrics and mental health indices. For instance, there was a moderate to strong positive relationship between the minimum value of accelerometer data along the x-axis (acl_X_min) and depression (ρ=0.410; *P*<.001) and anxiety (ρ=0.338; *P*=.003) scores. A significant negative relationship also emerged between the SD of accelerometer data along the x-, y-, and z- axes (acl_X_std, acl_Y_std, acl_Z_std) and depression (ρ=−0.314; *P*=.006; ρ=−0.309; *P*=.007; and ρ=−0.249; *P*=.03, respectively). A significant positive relationship was evident for anxiety scores, particularly strongly for minimum value along the y-axis (acl_Y_min) and GAD-7 scores (ρ=0.349; *P*=.002).

**Table 5:** Significant Spearman rank correlations between the infinite runner game metrics and Patient Health Questionnaire-8 (PHQ-8) and Generalized Anxiety Disorder-7 (GAD-7) scores.

Metric			Spearman rank correlation (ρ)		*P* value
**PHQ-8**					
	swipe_start_posY_std	−0.244		.03	
	swipe_end_press_mean	−0.278		.02	
	swipe_end_press_median	−0.300		.009	
	swipe_end_press_std^a^	−0.243		.04	
	swipe_end_press^b^_variance_mean	−0.405		<.001	
	swipe_end_press_variance_max	−0.303		.008	
	swipe_end_press_variance_median	−0.389		<.001	
	swipe_end_press_variance_std	−0.263		.02	
	swipe_end_press_variance_last	−0.336		.003	
	swipe_press_variance_mean	−0.242		.04	
	swipe_press_variance_min	−0.287		.01	
	swipe_speed_mean	−0.331		.003	
	swipe_speed_max	−0.282		.01	
	swipe_speed_median	−0.301		.008	
	swipe_speed_std	−0.257		.03	
	acl^c^_X_mean	0.257		.03	
	acl_X_median	0.265		.02	
	acl_X_std	−0.314		.006	
	acl_X_min	0.410		<.001	
	acl_X_range	−0.346		.002	
	acl_Y_std	−0.309		.007	
	acl_Y_min	0.255		.03	
	acl_Y_range	−0.323		.005	
	acl_Z_std	−0.249		.03	
	acl_Z_max	−0.280		.01	
	acl_Z_range	−0.295		.009	
**GAD-7**					
	swipe_count	−0.266		.02	
	swipe_start_posX_max	−0.239		.04	
	swipe_start_posY_std	−0.271		.02	
	swipe_end_press_mean	−0.235		.04	
	swipe_end_press_variance_mean	−0.309		.007	
	swipe_end_press_variance_max	−0.304		.008	
	swipe_press_variance_max	−0.245		.03	
	swipe_speed_mean	−0.272		.02	
	swipe_speed_std	−0.234		.04	
	time_between_swipes_std	0.274		.02	
	acl_X_std	−0.298		.009	
	acl_X_min	0.338		.003	
	acl_X_range	−0.294		.009	
	acl_Y_std	−0.272		.02	
	acl_Y_min	0.349		.002	
	acl_Y_range	−0.246		.03	
	acl_Z_std	−0.248		.03	
	acl_Z_max	−0.248		.03	

^a^press: pressure.

^b^std: SD.

^c^acl: accelerometer.

#### Object Slicing Game Analysis

Several metrics exhibited significant correlations with PHQ-8 and GAD-7 scores, as shown in Table 6. Notably, the minimum y-coordinate position when starting the swipes (swipe_start_posY_min) was significantly positively correlated with depression (ρ=0.368; *P*<.001) and anxiety (ρ=0.370; *P*<.001) scores. In addition, the minimum average swipe pressure also demonstrated a moderate positive correlation with depression (ρ=0.388; *P*<.001) and a moderate to strong positive correlation with anxiety (ρ=0.430; *P*<.001). Furthermore, accelerometer measurements were negatively associated with acl_Z_max, having the strongest negative association with both depression (ρ=−0.402; *P*<.001) and anxiety (ρ=−0.324; *P*=.004) scores.

**Table 6:** Significant Spearman rank correlations between the object slicing game metrics and the Patient Health Questionnaire-8 (PHQ-8) and Generalized Anxiety Disorder-7 (GAD-7) scores.

Metric		Spearman rank correlation (ρ)	*P* value
**PHQ-8**			
	swipe_start_posY_min	0.368	<.001
	swipe_start_posY_std	−0.244	.03
	swipe_end_posY_std^a^	−0.264	.01
	swipe_posY_std	−0.327	.005
	swipe_posY_min	0.270	.02
	swipe_posY_range	−0.286	.02
	swipe_press^b^_min	0.388	<.001
	swipe_press_variance_max	−0.235	.04
	swipe_duration_min	0.257	.02
	gyro_Y_range	−0.243	.03
	acl^c^_X_max	−0.282	.01
	acl_X_range	−0.290	.01
	acl_Y_min	0.281	.01
	acl_Z_std	−0.276	.02
	acl_Z_max	−0.402	<.001
	acl_Z_range	−0.378	<.001
**GAD-7**			
	swipe_start_posX_std	−0.233	.04
	swipe_start_posY_min	0.370	<.001
	swipe_start_posY_std	−0.27	.02
	swipe_end_posX_std	−0.242	.03
	swipe_end_posY_min	0.227	.046
	swipe_end_posY_std	−0.285	.01
	swipe_posX_std	−0.259	.03
	swipe_posX_min	0.237	.046
	swipe_posX_range	−0.236	.048
	swipe_posY_std	−0.364	.001
	swipe_posY_min	0.296	.01
	swipe_posY_range	−0.235	.049
	swipe_end_press_mean	0.231	.040
	swipe_press_min	0.430	<.001
	acl_Y_min	0.255	.03
	acl_Y_median	0.245	.03
	acl_Z_mean	−0.245	.03
	acl_Z_median	−0.256	.03
	acl_Z_std	−0.231	.04
	acl_Z_max	−0.324	.004
	acl_Z_range	−0.265	.02

^a^press: pressure.

^b^std: SD.

^c^acl: accelerometer.

## Discussion

### Principal Findings

#### Overview

In this exploratory study, we investigated the relationship between swipe gesture interactions and symptoms of anxiety and depression across 3 distinct mobile games. This study presents novel evidence and is among the first to examine how specific swipe gesture interactions from mobile games can act as potential indicators of anxiety and depression symptoms. Our findings revealed that swipe metrics such as swipe speed and swipe pressure were significantly associated with symptoms of anxiety and depression, as measured by the GAD-7 and PHQ-8 measures, respectively.

#### Psychological Interpretation

Anxiety and depressive disorders are linked to abnormalities in core cognitive and psychomotor processes. It is well known that these conditions are often associated with impairments in attention, decision-making, working memory, speed of information processing, and motor coordination. A central tenet of the attention control theory [52] is that anxiety can disrupt attentional control mechanisms, such as the ability to shift attention, inhibit irrelevant information, and maintain attentional focus that can subsequently negatively impact goal-oriented motor processing and working memory capacity. In the context of our findings, slower swipe speed observed among participants with higher anxiety scores may reflect difficulties with attentional control, as they may process and respond to in-game stimuli less efficiently. Furthermore, psychomotor slowing, known to be associated with depressive disorders, may contribute to the slower interactions within the games, as this involves a general slowing of both physical movements and cognitive processing and could therefore easily manifest as slower user-game interactions in the form of slower swipe speed.

Increased pressure during the swipes could be interpreted as an indicator of frustration or heightened muscle tension, both of which are physiological symptoms associated with anxiety disorders. The relationships between swipe pressure and mental health indices identified in this study elaborate on previous research on handwriting analysis, where heightened pen pressure during handwriting tasks was linked with anxiety and depression [53,54]. The novel findings from this study linking swipe pressure to symptoms of anxiety and depression opens up new avenues and suggests that the association between elevated pressure and mental health can be extended to subtle swipe gestures performed during mobile gaming. In addition, the device motion metrics recorded through an accelerometer were also significantly associated with anxiety and depression during the object slicing and infinite runner games. Therefore, the device’s motion could indicate symptoms such as lack of energy, fatigue, or heightened muscle tension commonly reported in individuals with anxiety and depression.

It is well-documented that attentional biases are a pervasive feature of anxiety disorders, with anxious individuals more likely to allocate disproportionate attention to perceived threats or particular stimuli [55]. In this study, an intriguing finding was in regard to the starting positions of the swipe gestures along the x- and y- axes, which were consistent and higher for those with elevated anxiety levels in the infinite runner and object slicing game but not in the puzzle game. For these two games, players need to avoid hitting the obstacles or avoid missing the objects resulting in a game over status. In contrast, the puzzle game lacked the same immediate threat of failure or time pressure to complete individual levels, offering the players enough time to plan their moves without the risk of an immediate game over status. It seems plausible that these differences in game mechanics, such as time pressure or immediate game over status, heighten the perceived threat of failure, eliciting an attentional bias that can lead to increased attentional fixation on specific locations of the screen leading to higher swipe positions observed during these games. However, given the exploratory nature of this study, further research is needed to validate these new findings.

Collectively, this study makes several notable contributions, with the identification of these novel metrics representing a substantial advancement in understanding how simple swipe gesture interactions during mobile gameplay can reflect underlying cognitive and behavioral responses associated with anxiety and depression.

#### Game Design Implications

If the long-term vision is to use mobile games as potential screening tools for anxiety and depressive disorders, it is crucial to understand what type of games are optimally effective or which game mechanics most consistently elicit behavioral responses indicative of symptoms of anxiety and depression. This was the reason we experimented with 3 games that differed substantively in their design and gameplay mechanics to identify whether one game genre or gameplay design is more effective than the other.

It is important to consider that mobile gameplay can introduce several confounding variables in the form of familiarity with mobile games, previous gaming skill and experience, or even age and gender. For instance, such mobile game genres as role-playing games or action and adventure games may not be ideal, as these often require complex controls, potentially skewing the data. In contrast, CMGs, such as the ones used in this study, rely on simple mechanics that are less likely to be influenced by gaming skills. However, there may be a case that more experienced mobile game players may have developed specific strategies that might make it harder to detect subtle anxiety and depression related behaviors. Therefore, an important next step in this literature is to investigate whether the observed metrics and their relationship with anxiety and depression are moderated by previous gaming experience and familiarity with the game.

The core element to consider in the design of mobile games is swipe gestures, as that is the most basic interaction. Our results indicated that various swipe gesture metrics, particularly swipe speed, swipe pressure, and swipe positions, were significantly related to anxiety and depression scores across all 3 games and may be independent from the game type and game mechanics. If this is the case, then many other types of touch interactions such as tapping and dragging should theoretically also be sensitive to these impairments. Furthermore, game interactions that involve pressure variability to dodge the obstacles or to jump higher or lower depending on the touch pressure, or those mechanics that require users to swipe faster or slower in response to in-game stimuli could provide additional insights into whether individuals tend to exhibit anxious or depressive tendencies as reflected in these interactions. Further research is the next important step to directly test these possibilities.

#### Ethical Challenges and Concerns

Despite the promising findings from this study on the potential of swipe gestures from mobile games as indicators of anxiety and depression, several important ethical considerations need to be acknowledged. As mentioned previously, data collection from mobile devices has been a part of growing debate regarding its privacy concerns [56-58]. Although mobile games pose fewer privacy concerns compared to other approaches, it is crucial that no personal or sensitive information is collected and that the collected data are anonymized. A further key ethical challenge lies in ensuring that any communication of mental health information generated from the mobile gameplay is done so in a manner that does not elicit distress. Any insights or potential indications should be communicated carefully, providing users with appropriate guidance and resources to seek professional help. The feedback from the mobile games should be clearly framed as nondiagnostic and encourage users to consult a trained mental health professional for a comprehensive assessment.

While CMGs offer a nonintrusive, engaging approach using only virtual data generated within gaming worlds, they also have the potential to be misused. For instance, commercial companies might use these data to target susceptible individuals with manipulative strategies, such as in-game purchases to players exhibiting impulsivity disorders like obsessive compulsive disorders, because they are likely to buy more. As mentioned earlier, game analytics has been predominantly used for monetization benefits. Therefore, companies could dynamically adjust game mechanics to exploit the end users’ mental health vulnerabilities, encouraging addictive gameplay behaviors or excessive spending.

Therefore, ethical safeguards surrounding the potential use of CMGs to track mental health symptoms must be enforced to address these ethical concerns. It is important to underscore that swipe gesture interactions or any other data provided by mobile games in general seem to offer the greatest promise with several advantages, including easy accessibility, scalability, and a noninvasive and highly engaging screening tool, but are not a replacement for traditional clinical assessment.

### Limitations and Future Work

This study provides valuable insights into the relationship between swipe gesture interactions from mobile games and symptoms of anxiety and depression, but several limitations of this study also need to be acknowledged. First, the sample size of 82 participants, while adequate for exploratory analysis and preliminary findings, may limit the generalizability of the results to the broader population. Our sample consisted predominantly of young adults, with 95% (n=78) of participants aged between 18 and 30 years, while only 5% (n=4) were aged between 31 and 40 years, as shown in Table 2. Consequently, the findings may not generalize to older adults. Future studies should aim to recruit larger and more diverse samples from different demographic characteristics and varied gaming behavior groups to cross-validate the findings.

Second, the presence of an instructor with the participants when they played the games introduces the possibility of a potential observer effect. Finally, in this study, we did not test whether there were potential demographic and gaming behavior differences that might influence the swipe gesture patterns, and future work should explore how demographic factors, such as age and gender as well as gaming habits, influence these interactions.

In addition, the study was conducted in a controlled environment where participants followed a strict protocol of holding the device in one hand and playing the game with the other hand using only a single finger touch. While this approach helped to minimize the effect of confounding variables, it may not reflect participants’ natural behavior when playing mobile games. Therefore, future studies are needed to assess these interactions in a natural setting where participants can play mobile games without any protocol.

Another limitation is that no correction for multiple comparisons was applied to the correlation analysis. This was because of the preliminary nature of this study, which was designed simply to identify potentially relevant behavioral metrics for future investigation. However, future confirmatory studies based on these findings, especially those with prespecified hypotheses and smaller feature sets, constitute the next important contribution to the literature in this field of study.

Future research could also expand the findings from this study to include gameplay performance metrics such as maximum score, total levels cleared, time taken to clear levels, total obstacles hit, or maximum distance run. Combining gameplay performance metrics with swipe gesture metrics could provide a more comprehensive understanding of how anxiety and depression symptoms manifest within the mobile gaming contexts. We used event-based data collection where swipe metrics were recorded for each swipe event during the gameplay. While this approach helped prevent any framerate drops and overcollection of data, it may overlook granular swipe gesture patterns. Therefore, future work should consider sampling data more frequently based on smaller time intervals to capture more detailed and fine-grained insights into swipe and gameplay behaviors. Finally, the findings from this study also set a precedent for future work involving machine learning algorithms to develop predictive models using mobile gameplay data that could potentially predict anxiety and depression scores.

### Conclusions

This study reports novel data that speak to the potential value of swipe gesture interactions from mobile games as indicators of symptoms of anxiety and depression. To the best of our knowledge, this is the first study to explore the relationship between swipe gesture metrics and validated measures of anxiety and depression. Across 3 distinct mobile games (a puzzle game, an infinite runner game, and an object slicing game), the results revealed significant associations between swipe gesture metrics that included swipe speed, swipe pressure variability, and swipe starting positions with both anxiety and depression. These new insights can be supported by the broader mental health literature that shows both depression and anxiety are associated with abnormalities in core psychomotor and cognitive processes as well as attentional biases. In conclusion, this study makes a significant contribution by presenting important foundational evidence on the potential of swipe gesture interactions from mobile games as a novel method for screening symptoms of anxiety and depression.

## Appendix

Multimedia Appendix 1 A visual depiction of the swipes performed during each of the 3 games.

Multimedia Appendix 2 List of all metrics used in the analysis and their definitions.

## Abbreviations

ASD: autism spectrum disorder
CMG: casual mobile game
GAD-7: Generalized Anxiety Disorder-7
JSON: JavaScript Object Notation
PHQ-8: Patient Health Questionnaire-8
