# Supplementary material for: Leveraging Swipe Gesture Interactions From Mobile Games as Indicators of Anxiety and Depression: Exploratory Study
Source: JMIR Ment Health. 2025 Jun 26;12:e70577. doi: 10.2196/70577 (PMC12246760; doi:10.2196/70577)

A visual depiction of swipes performed by a random user (“user_23”) in random game levels (“game_round_ID”) each of the three games: (a) Infinite Runner Game (b) Object-Slicing Game (c) Puzzle Game.


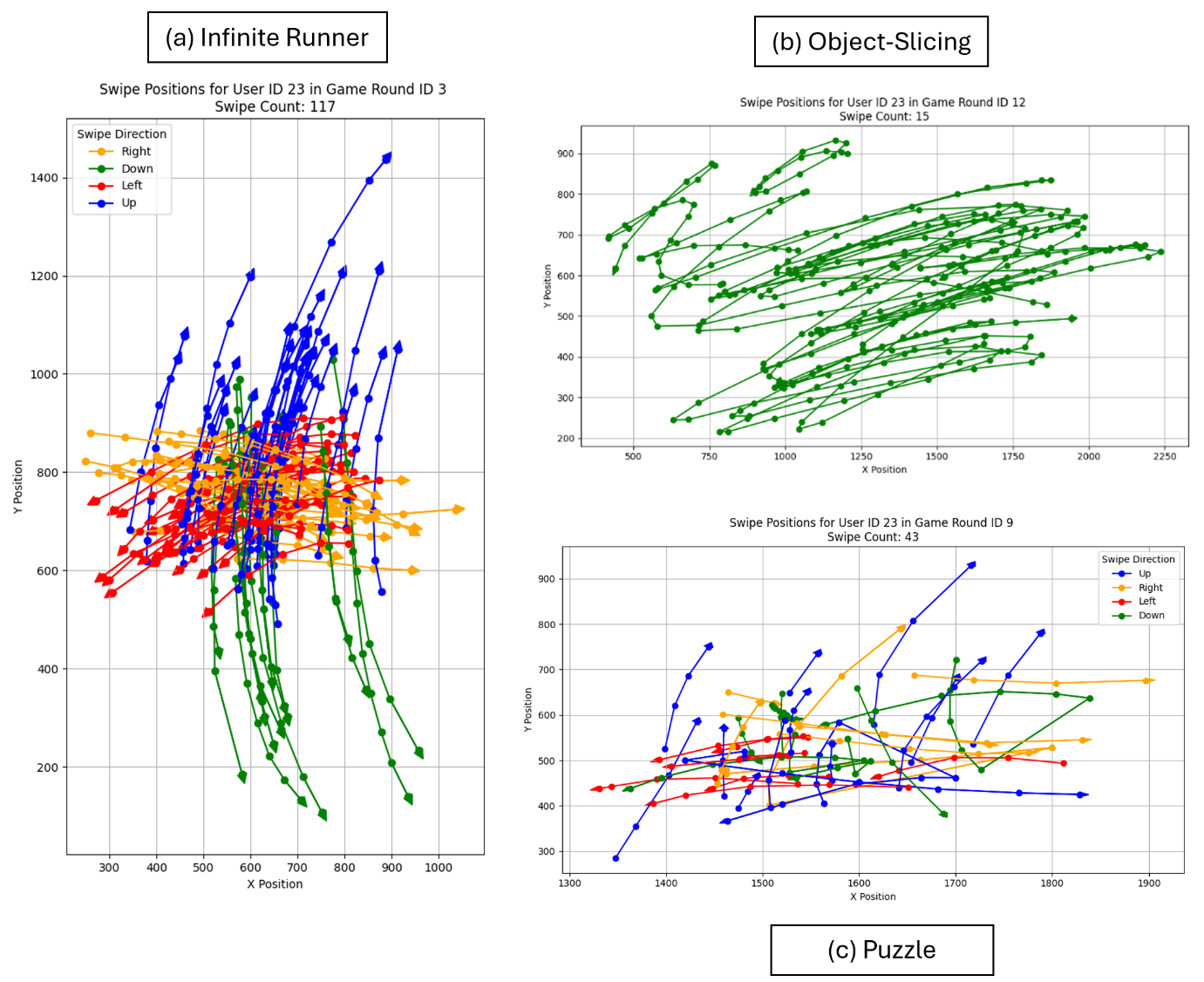

Supplement: Multimedia Appendix 1 [file mental_v12i1e70577_app1.docx]
