# Supplementary material for: Leveraging Swipe Gesture Interactions From Mobile Games as Indicators of Anxiety and Depression: Exploratory Study
Source: JMIR Ment Health. 2025 Jun 26;12:e70577. doi: 10.2196/70577 (PMC12246760; doi:10.2196/70577)
Supplement: Multimedia Appendix 2 [file mental_v12i1e70577_app2.docx]

List of all metrics including their definitions. The aggregation is done for the entire session for each game of the three games separately.

| **Metric** | **Description** |
| --- | --- |
| swipe_ID_count | Total swipes performed by the user during the entire session |
| swipe_ID_mean | The average number of swipes performed by the user during the entire session.  This is calculated as a ratio of total swipes to total levels completed |
| swipe_ID_std | The variance in total swipes performed at each level during the entire session |
| swipe_event_time | The timestamp of current swipe event. Calculated from when the game session began. |
| swipe_start_time | The timestamp of when the current swipe was initiated. Calculated from when the game level was launched. |
| swipe_end_time | The timestamp of when the current swipe was completed. Calculated from when the game level was launched. |
| **Swipe Start Position (X and Y screen coordinates)** | |
| swipe_start_posX_mean | Mean horizontal (X-axis) screen coordinate at which swipes began across the session. |
| swipe_start_posX_median | Median of swipe start positions along the X-axis. |
| swipe_start_posX_max | Maximum horizontal (X-axis) screen coordinate at which any swipe began during the entire session. |
| swipe_start_posX_min | Minimum horizontal (X-axis) screen coordinate at which any swipe began during the entire session. |
| swipe_start_posX_std | Standard deviation of swipe start positions along the X-axis. |
| swipe_start_posX_range | Difference between max and min swipe start positions along X-axis. |
| swipe_start_posX_first | X-axis screen coordinate position when the very first swipe was initiated. |
| swipe_start_posX_last | X-axis screen coordinate position when the final swipe was initiated. |
|  |  |
| swipe_start_posY_mean | Mean vertical (Y-axis) screen coordinate at which swipes began across the session. |
| swipe_start_posY_median | Median of swipe start positions along the Y-axis. |
| swipe_start_posY_max | Maximum vertical (Y-axis) screen coordinate at which any swipe began during the entire session. |
| swipe_start_posY_min | Minimum vertical (Y-axis) screen coordinate at which any swipe began during the entire session. |
| swipe_start_posY_std | Standard deviation of swipe start positions along the Y-axis. |
| swipe_start_posY_range | Difference between max and min swipe start positions along Y-axis. |
| swipe_start_posY_first | Y-axis screen coordinate position when the very first swipe was initiated. |
| swipe_start_posY_last | Y-axis screen coordinate position when the final swipe was initiated. |
| **Swipe End Position (X and Y screen coordinates)** | |
| swipe_end_posX_mean | Mean horizontal (X-axis) screen coordinate at which swipes were completed across the session. |
| swipe_end_posX_median | Median of swipe completion positions along the X-axis. |
| swipe_end_posX_max | Maximum horizontal (X-axis) screen coordinate at which any swipe was completed during the entire session. |
| swipe_end_posX_min | Minimum horizontal (X-axis) screen coordinate at which any swipe was completed during the entire session. |
| swipe_end_posX_std | Standard deviation of swipe completion positions along the X-axis. |
| swipe_end_posX_range | Difference between max and min swipe completion positions along X-axis. |
| swipe_end_posX_first | X-axis screen coordinate position when the very first swipe was completed. |
| swipe_end_posX_last | X-axis screen coordinate position when the final swipe was completed. |
| swipe_end_posY_mean | Mean vertical (Y-axis) screen coordinate at which swipes were completed across the session. |
| swipe_end_posY_median | Median of swipe completion positions along the Y-axis. |
| swipe_end_posY_max | Maximum vertical (Y-axis) screen coordinate at which any swipe was completed during the entire session. |
| swipe_end_posY_min | Minimum vertical (Y-axis) screen coordinate at which any swipe was completed during the entire session. |
| swipe_end_posY_std | Standard deviation of swipe completion positions along the Y-axis. |
| swipe_end_posY_range | Difference between max and min swipe completion positions along Y-axis. |
| swipe_end_posY_first | Y-axis screen coordinate position when the very first swipe was completed. |
| swipe_end_posY_last | Y-axis screen coordinate position when the final swipe was completed. |
| **Swipe Position (List of all X and Y coordinates for the current swipe path)** | |
| swipe_posX_mean | The mean of all horizontal (X-axis) screen coordinate samples recorded along each swipe path across the entire session. |
| swipe_posX_median | Median of all horizontal (X-axis) coordinate samples recorded along every swipe path across the entire session. |
| swipe_posX_max | Maximum horizontal (X-axis) screen coordinate reached at any point along all swipe paths across the entire session. |
| swipe_posX_min | Minimum horizontal (X-axis) screen coordinate reached at any point along all swipe paths across the entire session. |
| swipe_posX_std | Standard deviation of all horizontal coordinate samples across swipe paths. |
| swipe_posX_range | Difference between max and min swipe positions along X-axis across all swipe paths. |
|  |  |
| swipe_posY_mean | The mean of all vertical (Y-axis) screen coordinate samples recorded along each swipe path across the entire session. |
| swipe_posY_median | Median of all vertical (Y-axis) coordinate samples recorded along every swipe path across the entire session. |
| swipe_posY_max | Maximum vertical (Y-axis) screen coordinate reached at any point along all swipe paths across the entire session. |
| swipe_posY_min | Minimum vertical (Y-axis) screen coordinate reached at any point along all swipe paths across the entire session. |
| swipe_posY_std | Standard deviation of all vertical coordinate samples across swipe paths. |
| swipe_posY_range | Difference between max and min swipe positions along Y-axis across all swipe paths. |
| **Swipe Start Pressure** | |
| swipe_start_press_mean | Mean touch pressure value recorded at the moment each swipe began, averaged across all swipes in the session. |
| swipe_start_press_median | Median touch pressure value recorded at the start of each swipe, across the session. |
| swipe_start_press_max | Maximum touch pressure value recorded at the start of any swipe, across the entire session |
| swipe_start_press_min | Minimum touch pressure value recorded at the start of any swipe, across the entire session |
| swipe_start_press_std | Standard deviation of touch pressure values at the moment each swipe began, across all swipes during the entire session. |
| swipe_start_press_range | Difference between the maximum and minimum touch pressure values at swipe start. |
| swipe_start_press_first | Touch pressure value recorded at the start of the very first swipe in the session. |
| swipe_start_press_last | Touch pressure value recorded at the start of the final swipe in the session. |
| **Swipe End Pressure** | |
| swipe_end_press_mean | Mean touch pressure value recorded at the moment each swipe ended, averaged across all swipes in the session. |
| swipe_end_press_median | Median touch pressure value recorded at the end of each swipe, across the session. |
| swipe_end_press_max | Maximum touch pressure value recorded at the end of any swipe, across the entire session |
| swipe_end_press_min | Minimum touch pressure value recorded at the end of any swipe, across the entire session |
| swipe_end_press_std | Standard deviation of touch pressure values at the moment each swipe ended, across all swipes during the entire session. |
| swipe_end_press_range | Difference between the maximum and minimum touch pressure values at swipe completion. |
| swipe_end_press_first | Touch pressure value recorded at the end of the very first swipe in the session. |
| swipe_end_press_last | Touch pressure value recorded at the end of the final swipe in the session. |
| **Swipe Start and Swipe End Pressure Variance** | |
| swipe_start_press_variance_mean | Mean of variance in touch pressure values at the moment each swipe began, across the session. |
| swipe_start_press_variance_ median | Median of variance in touch pressure values recorded at the start of each swipe, across the session. |
| swipe_start_press_variance_max | The maximum variance in touch pressure values recorded at the start of any swipe, across the entire session |
| swipe_start_press_variance_min | The minimum variance in touch pressure values recorded at the start of any swipe, across the entire session |
| swipe_start_press_variance_std | The standard deviation of variance in touch pressure values at the moment each swipe began, across all swipes. |
| swipe_start_press_variance_range | The difference between the maximum and minimum variance in touch pressure values at swipe start. |
| swipe_start_press_variance_first | The variance in touch pressure value recorded at the start of the very first swipe in the session. |
| swipe_start_press_variance_last | The variance in touch pressure value recorded at the start of the final swipe in the session. |
| swipe_end_press_variance_mean | Mean of variance in touch pressure values at the moment each swipe ended, averaged across all swipes in the session. |
| swipe_end_press_variance_ median | Median of variance in touch pressure values recorded at the end of each swipe, across the session. |
| swipe_end_press_variance_max | The maximum variance in touch pressure values recorded at the end of any swipe, across the entire session |
| swipe_end_press_variance_min | The minimum variance in touch pressure values recorded at the end of any swipe, across the entire session |
| swipe_end_press_variance_std | The standard deviation of variance in touch pressure values at the moment each swipe ended, across all swipes. |
| swipe_end_press_variance_range | The difference between the maximum and minimum variance in touch pressure values at swipe completion. |
| swipe_end_press_variance_first | The variance in touch pressure values recorded at the end of the very first swipe in the session. |
| swipe_end_press_variance_last | The variance in touch pressure values recorded at the end of the final swipe in the session. |
| **Swipe Pressure**  **(List of all touch pressure values for the current swipe)** | |
| swipe_press_mean | Mean of all touch pressure samples recorded along each swipe path, across the entire session |
| swipe_press_median | Median of all touch pressure samples recorded along each swipe path, across the entire session. |
| swipe_press_max | Maximum touch pressure value recorded at any point along all swipe paths in the session. |
| swipe_press_min | Minimum touch pressure value recorded at any point along all swipe paths in the session. |
| swipe_press_std | Standard deviation of touch pressure samples across all swipe paths. |
| swipe_press_range | Difference between the maximum and minimum touch pressure values across all swipe paths. |
| **Swipe Pressure Variance**  **(The variance in touch pressure recorded for the duration current swipe)** | |
| swipe_press_variance_mean | Mean of variance in touch pressure values throughout each swipe, across all swipes in the session. |
| swipe_press_variance_median | Median of variance in touch pressure values throughout each swipe, across the session. |
| swipe_press_variance_max | The maximum variance in touch pressure values within any swipe, across the entire session |
| swipe_press_variance_min | The minimum variance in touch pressure values within any swipe, across the entire session |
| swipe_press_variance_std | The standard deviation of variance in touch pressure values across all swipes. |
| swipe_press_variance_range | The difference between the maximum and minimum variance in touch pressure values. |
| swipe_press_variance_first | The variance in touch pressure value during the first swipe in the session. |
| swipe_press_variance_last | The variance in touch pressure value during the final swipe in the session. |
| **Swipe Speed** | |
| swipe_speed_mean | The mean speed (in pixels per seconds) of all swipes across the entire session. |
| swipe_speed_median | The median speed of all swipes across the entire session. |
| swipe_speed_max | The maximum speed recorded for any swipe during the entire session. |
| swipe_speed_min | The minimum speed recorded for any swipe during the entire session. |
| swipe_speed_std | The standard deviation of swipe speeds of all swipes across the entire session. |
| swipe_speed_range | Difference between the maximum and minimum swipe speeds in the session. |
| swipe_speed_first | The speed of the very first swipe executed in the session. |
| swipe_speed_last | The speed of the final swipe executed in the session. |
| **Swipe Duration** | |
| swipe_duration_mean | The mean time (s) taken to complete a swipe across the entire session. |
| swipe_duration_median | The median time taken to complete a swipe across the entire session. |
| swipe_duration_max | The maximum time taken to complete any single swipe during the entire session. |
| swipe_duration_min | The minimum time taken to complete any single swipe during the entire session. |
| swipe_duration_std | Standard deviation of swipe durations across all swipes. |
| swipe_duration_range | Difference between the maximum and minimum swipe durations in the session. |
| swipe_duration_first | Time taken to complete the very first swipe in the session. |
| swipe_duration_last | Time taken to complete the final swipe in the session. |
| **Swipe Distance** | |
| swipe_distance_mean | Mean distance (in pixels) covered during a swipe across the entire session. |
| swipe_distance_median | Median distance (in pixels) covered during a swipe across the entire session. |
| swipe_distance_max | Maximum distance covered by any single swipe during the session. |
| swipe_distance_min | Minimum distance covered by any single swipe during the session. |
| swipe_distance_std | Standard deviation of swipe distances across all swipes during the session. |
| swipe_distance_range | Difference between the maximum and minimum swipe distances in the session. |
| swipe_distance_first | Distance covered by the very first swipe in the session. |
| swipe_distance_last | Distance covered by the final swipe in the session. |
| **Time Between Swipes** | |
| time_between_swipes_mean | The mean time interval (s) between consecutive swipes, across the session. |
| time_between_swipes_median | The median time interval (s) between swipes, across the session |
| time_between_swipes_max | The maximum time interval (s) between any two consecutive swipes across the session. |
| time_between_swipes_min | The minimum time interval (s) between any two consecutive swipes across the session. |
| time_between_swipes_std | Standard deviation of inter-swipe intervals across the session. |
| time_between_swipes_range | Difference between the maximum and minimum inter-swipe intervals in the session. |
| time_between_swipes_first | Time interval (s) between the first and second swipe in the session. |
| time_between_swipes_last | Time interval (s) between the last two swipes in the session. |
| **Accelerometer Readings across X, Y and Z-axes** | |
| acl_X_mean | Mean acceleration recorded along the device’s X-axis (side-to-side/lateral) across all time samples in the session. |
| acl_X_median | Median acceleration along the X-axis across the session. |
| acl_X_max | Maximum acceleration value recorded on the X-axis. |
| acl_X_min | Minimum acceleration value recorded on the X-axis. |
| acl_X_std | Standard deviation of X-axis acceleration samples across the session. |
| acl_X_range | Difference between the maximum and minimum X-axis acceleration values across the session. |
| acl_Y_mean | Mean acceleration recorded along the device’s Y-axis (up-down/vertical) across all time samples in the session. |
| acl_Y_median | Median acceleration along the Y-axis across the session. |
| acl_Y_max | Maximum acceleration value recorded on the Y-axis. |
| acl_Y_min | Minimum acceleration value recorded on the Y-axis. |
| acl_Y_std | Standard deviation of Y-axis acceleration samples across the session. |
| acl_Y_range | Difference between the maximum and minimum Y-axis acceleration values across the session. |
| acl_Z_mean | Mean acceleration recorded along the device’s Z-axis (front-back/depth) across all time samples in the session. |
| acl_Z_median | Median acceleration along the Z-axis across the session. |
| acl_Z_max | Maximum acceleration value recorded on the Z-axis. |
| acl_Z_min | Minimum acceleration value recorded on the Z-axis. |
| acl_Z_std | Standard deviation of Z-axis acceleration samples across the session. |
| acl_Z_range | Difference between the maximum and minimum Z-axis acceleration values across the session. |
| **Gyroscope Readings across X, Y, Z and W components** | |
| gyro_X_mean | Mean angular velocity around the device’s X-axis (pitch) across all time samples in the session. |
| gyro_X_median | Median angular velocity around the X-axis across the session. |
| gyro_X_max | Maximum angular velocity recorded on the X-axis. |
| gyro_X_min | Minimum angular velocity recorded on the X-axis. |
| gyro_X_std | Standard deviation of angular velocity around the X-axis across all time samples. |
| gyro_X_range | Difference between the maximum and minimum X-axis angular velocity values. |
| gyro_Y_mean | Mean angular velocity around the device’s Y-axis (roll) across all time samples in the session. |
| gyro_Y_median | Median angular velocity around the Y-axis across the session. |
| gyro_Y_max | Maximum angular velocity recorded on the Y-axis. |
| gyro_Y_min | Minimum angular velocity recorded on the Y-axis. |
| gyro_Y_std | Standard deviation of angular velocity around the Y-axis across all time samples. |
| gyro_Y_range | Difference between the maximum and minimum Y-axis angular velocity values. |
| gyro_Z_mean | Mean angular velocity around the device’s Z-axis (yaw) across all time samples in the session. |
| gyro_Z_median | Median angular velocity around the Z-axis across the session. |
| gyro_Z_max | Maximum angular velocity recorded on the Z-axis. |
| gyro_Z_min | Minimum angular velocity recorded on the Z-axis. |
| gyro_Z_std | Standard deviation of angular velocity around the Z-axis across all time samples. |
| gyro_Z_range | Difference between the maximum and minimum Z-axis angular velocity values. |
| gyro_W_mean | Mean quaternion W component across all time samples, summarizing overall device orientation stability. |
| gyro_W_median | Median quaternion W component across the session. |
| gyro_W_max | Maximum quaternion W component recorded. |
| gyro_W_min | Minimum quaternion W component recorded. |
| gyro_W_std | Standard deviation of quaternion W across all time samples. |
| gyro_W_range | Difference between the maximum and minimum quaternion W values. |
